# Hemorrhagic Longitudinally Extensive Transverse Myelitis

**DOI:** 10.1155/2016/1596864

**Published:** 2016-10-25

**Authors:** Chris Y. Wu, Tanawan Riangwiwat, Beau K. Nakamoto

**Affiliations:** ^1^Department of Medicine, University of Hawaii, Honolulu, HI, USA; ^2^Department of Neurology, Straub Clinics and Hospital, Honolulu, HI, USA

## Abstract

Longitudinally extensive transverse myelitis (LETM) may be associated with viral triggers, including both infections and vaccinations. We present a case of a healthy immunocompetent 33-year-old woman who developed a hemorrhagic LETM 2 weeks after seasonal influenza vaccination. Hemorrhagic LETM has not to our knowledge been reported after influenza vaccination. It may represent a forme fruste variant of acute hemorrhagic leukoencephalitis.

## 1. Introduction

Central nervous system demyelinating diseases can rarely present after viral infections or vaccinations [1–3]. We present a case of hemorrhagic longitudinally extensive transverse myelitis in a healthy, young, and immunocompetent patient after seasonal influenza vaccination.

## 2. Case Report

A 33-year-old Japanese woman with no past medical history was admitted for acute low back pain, asymmetric lower extremity (LE) numbness and paraparesis, and urinary retention. Symptoms had been worsening for 4 days. She had presented to the emergency department 3 days before with milder symptoms but was discharged after radiographs and computed tomography (CT) scans of her thoracic and lumbar spine were normal. Additionally, she had slipped and fell on her buttocks 2 weeks ago with no sequelae. Bladder scan on admission showed 800 cm^3^ of urine. The patient denied any recent cough or colds but did receive a trivalent inactivated seasonal influenza vaccine 2 weeks prior to the onset of symptoms. She had no family history of neurologic diseases and had no recent travel history.

**Figure 1:**
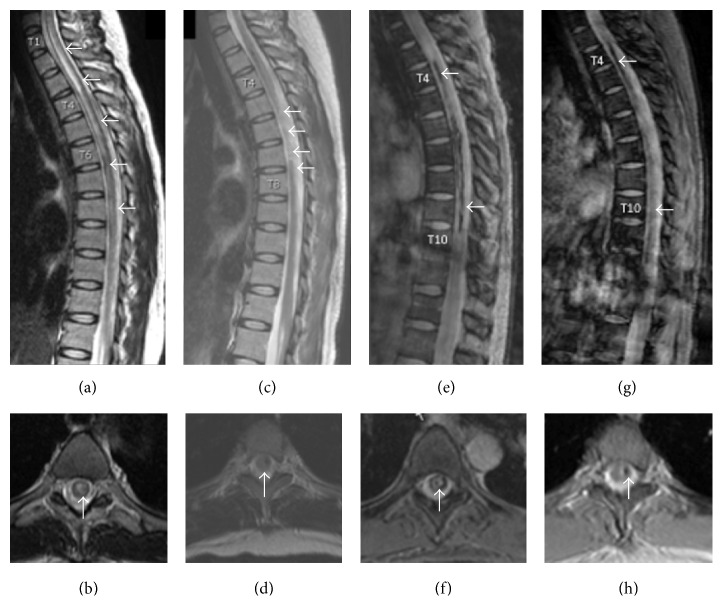
Cervical and thoracic MRI. At initial presentation, sagittal T2-weighted image (a) demonstrated extensive T2-signal abnormality from C6-C7 to T11-T12 involving the central gray matter (b). Sagittal gradient echo (GRE) showed decreased GRE signal extending from T3-T4 to T9-T10 (e, f). At three-month follow-up, the cord signal on T2-weighted imaging was normal (d) with volume loss within the cord from T3-T4 to T7-T8 (c, arrows). The long regions of decreased GRE signal within the central aspect of the cord compatible with hemosiderin deposition persisted (g, h). There was poorly defined abnormal enhancement within the cord behind the T5 and T6 vertebral bodies (not shown).

Vital signs on admission were within the normal range. Magnetic resonance imaging (MRI) of the cervical and thoracic spine with and without contrast showed extensive T2-signal abnormality extending from C7 to T11 primarily involving the central gray matter (Figures 1(a) and 1(b)) with diffuse enlargement of the involved cord. There was also abnormally decreased Gradient-Recalled-Echo (GRE) signal extending from T3-T4 to T9-T10 (Figures 1(e) and 1(f)). There was mildly increased T1-signal in this region on sagittal imaging. Axial imaging confirmed the abnormal increased T1-signal and decreased GRE signal on axial imaging compatible with hemorrhage within the substance of the cord. Postcontrast sequences did not demonstrate a mass-like enhancement. There were no flow voids along the cord. Neurologic exam was significant for asymmetric paraparesis with inability to move the left leg, while the right leg showed diminished motor strength (3 for right hip flexion, 4+ for knee extension, 4− for ankle dorsiflexion, and 5 for ankle plantar flexion). Reflexes were absent in the legs with upgoing Babinski reflexes bilaterally. The patient had a temperature sensory level of T4 but had normal vibratory sensation.

Despite intravenous methylprednisolone 1 g daily for 5 consecutive days followed by immunoglobulin therapy (IVIG) 2 g/kg divided over 5 days, the patient's LE weakness progressed to complete flaccid paraplegia. On lumbar puncture, cerebrospinal fluid (CSF) was hazy and colorless without xanthochromia. There were 55/*μ*L white blood cells with mild lymphocytic predominance, 2050/*μ*L red blood cells, 54 mg/dL of glucose, and 33 mg/dL of protein. Brain MRI and magnetic resonance angiography (MRA) of the brain and neck were both normal. Workup was negative for neuromyelitis optica [NMO] (negative CSF anti-NMO IgG, negative serum NMO/AQP-4 IgG), varicella zoster virus (VZV), enterovirus, herpes simplex viruses (HSV) 1 and 2, human T-cell lymphotropic viruses I and II, HIV, syphilis, B12 or folate deficiency, sarcoidosis, vertebral dissection, multiple sclerosis (negative CSF IgG index and oligoclonal bands), acute disseminating encephalomyelitis (normal brain MRI), spinal arteriovenous malformation/cavernoma and dural arteriovenous fistula, inherited hypercoagulopathies, cryoglobulinemia, and systemic autoimmune diseases. Copper (66 *μ*g/dL) and zinc (52 *μ*g/dL) levels were both mildly low. After 3 months, the patient had minimal improvement of her paraplegia. Repeat cervical and thoracic MRI with and without contrast showed resolution of edema and cord expansion, volume loss, and hemosiderin staining in the thoracic spinal cord (Figures 1(c), 1(d), 1(g), and 1(h)).

## 3. Discussion

We presented a patient who developed hemorrhagic LETM characterized by a longitudinally extensive T2-hyperintensity on cervical-thoracic MRI. RBC in the CSF and low signal intensity on GRE MR sequences proved spinal cord hemorrhage in this case. Potential causes of longitudinally extensive myelitis are heterogeneous, with neuromyelitis optica (NMO) being the most common (however, our patient did not fulfill diagnostic criteria due to negative CSF and serum NMO serologies, normal brain MRI, and absence of optic neuritis) [4]. A variety of other autoimmune, demyelinating, infectious, vascular, and neoplastic etiologies exist [4]. Although our workup was positive for copper deficiency, this is a rare cause of myelitis and has a subacute presentation and usually affects dorsal parts of the spinal cord [4]. Additionally, although we did not obtain a spinal angiogram to definitively rule out a spinal arteriovenous shunt, we did consult an interventional neuroradiologist, who assured us that our patient's history and imaging was inconsistent with spinal arteriovenous shunts (which usually has a subacute presentation with imaging findings in a specific vascular distribution), and as a result such a procedure was unnecessary [4]. Hemorrhagic LETM is uncommon and has been reported in associated with VZV and HSV-2 infections in immunocompromised and less commonly immunocompetent individuals [5–7]. Pathologic studies have demonstrated vasculitis, necrosis, and demyelination in VZV myelitis suggesting that hemorrhagic LETM in these cases may be the result of a small vessel vasculitis [5, 8]. Our patient was HIV-seronegative, and CSF studies were negative for VZV and HSV 1 and 2.

Our patient did receive a seasonal influenza vaccination 2 weeks prior to the onset of neurological symptoms. Although rare, vaccination has been temporally associated with devastating neurologic syndromes, including LETM and acute disseminated encephalomyelitis (ADEM) [1–3]. A recent case report presented a patient who developed LETM after vaccination with nasal attenuated novel influenza A (H1N1) vaccination [2]. Imaging findings were similar to our case, consisting of intramedullary nonenhancing T2 hyperintensity extending from the cervical medullary junction throughout the length of the thoracic cord. Though, in that case, the patient made a significant recovery after treatment with corticosteroids and plasmapheresis. Additionally, the authors did not report results of GRE MRI, and as a result, it is unknown if their case also included intramedullary hemorrhage. Association of LETM with influenza vaccination in this case is possible but not certain.

Hemorrhagic conversion of myelitis, or of encephalomyelitis, is uncommon. It has been reported to occur in acute hemorrhagic leukoencephalopathy (AHLE), a rare fulminant subtype of ADEM. AHLE usually involves the brain, but spinal cord involvement has been reported [3]. Additionally, it has been reported to occur in association with influenza infection [9]. In AHLE, an autoimmune response triggered by infectious, or other unknown agents, causes vascular injury and perivascular demyelination, leading to hemorrhage and necrosis surrounding small parenchymal vessels [9]. Histopathologic specimens show extensive perivascular demyelination, fibrinoid vascular necrosis, widespread perivascular mixed inflammatory infiltrate, and “ring and ball” hemorrhages [3]. Given that both influenza virus infection and influenza vaccination have been associated with ADEM, we hypothesize that our patient may have developed a variant of AHLE isolated to the spinal cord [3]. As such, her prognosis was poor due to the necrotizing pathology. An autoimmune pathophysiology is likely, possibly due to cross-reactivity between infectious agents and CNS targets [3]. Genetic susceptibility based on MHC haplotypes may also be involved in the immune response [3].

Central nervous system demyelinating diseases have been temporally associated with influenza vaccination [1–3]. We presented a case of hemorrhagic LETM, which may be a variant of hemorrhagic ADEM. Prognosis in our case was poor. More research is needed to identify susceptible individuals.
